# 3D-Printable PLA/Mg Composite Filaments for Potential Bone Tissue Engineering Applications

**DOI:** 10.3390/polym15112572

**Published:** 2023-06-03

**Authors:** Sumama Nuthana Kalva, Fawad Ali, Carlos A. Velasquez, Muammer Koç

**Affiliations:** 1 Division of Sustainable Development, College of Science and Engineering, Hamad Bin Khalifa University, Qatar Foundation, Doha P.O. Box 34110, Qatar; 2 Surgical Research Section, Innovation Unit, Hamad Medical Corporation, Doha P.O. Box 3050, Qatar

**Keywords:** 3D printing, magnesium, composite, PLA, bone implants

## Abstract

Magnesium (Mg) is a promising material for bone tissue engineering applications due to it having similar mechanical properties to bones, biocompatibility, and biodegradability. The primary goal of this study is to investigate the potential of using solvent-casted polylactic acid (PLA) loaded Mg (WE43) composites as filament feedstock for fused deposition modeling (FDM) 3D Printing. Four PLA/Magnesium (WE43) compositions (5, 10, 15, 20 wt%) are synthesized and produced into filaments, then used to print test samples on an FDM 3D printer. Assessments are made on how Mg incorporation affected PLA’s thermal, physicochemical, and printability characteristics. The SEM study of the films shows that the Mg particles are uniformly distributed in all the compositions. The FTIR results indicate that the Mg particles blend well with the polymer matrix and there is no chemical reaction between the PLA and the Mg particles during the blending process. The thermal studies show that the addition of Mg leads to a small increase in the melting peak reaching a maximum of 172.8 °C for 20% Mg samples. However, there are no dramatic variations in the degree of crystallinity among the Mg-loaded samples. The filament cross-section images show that the distribution of Mg particles is uniform up to a concentration of 15% Mg. Beyond that, non-uniform distribution and an increase in pores in the vicinity of the Mg particles is shown to affect their printability. Overall, 5% and 10% Mg composite filaments were printable and have the potential to be used as composite biomaterials for 3D-printed bone implants.

## 1. Introduction

Bone tissue engineering is an interdisciplinary field that aims to develop artificial bone substitutes that can replace or repair damaged bone tissues. The need for innovative materials and fabrication techniques to meet the medical requirements of biodegradability, biocompatibility, and on-demand customizable design and manufacture at cheap cost has resulted from the sharp increase in orthopedic implants. As a substitute for real bones, implants should mimic bone as closely as feasible in terms of strength, hardness, chemical composition, rate of deterioration, biocompatibility in the physiological environment, etc. [1,2]. Due to their great mechanical strength, biocompatibility, and beneficial corrosion resistance features, metals and metallic alloys, such as titanium, stainless steel, and Co-Cr alloys, make up the most commercially available implants [3,4,5,6]. The use of 3D-printing technology has revolutionized bone tissue engineering, providing a precise and customizable method for producing complex three-dimensional structures that mimic the natural architecture of bone [7]. Among the various materials used for 3D printing, polylactic acid (PLA) is a biocompatible and biodegradable polymer widely used for tissue engineering applications due to its excellent mechanical and biological properties [8].

However, PLA alone may not be sufficient for bone tissue engineering applications, as it lacks the necessary strength and toughness required for load-bearing bones [9]. Magnesium (Mg), on the other hand, is a biodegradable and bioactive metal that has shown great potential as a bone substitute due to its mechanical properties and ability to stimulate bone regeneration [10,11]. Combining PLA and Mg in a composite material could potentially create a 3D-printable filament that possesses both the biocompatibility and biodegradability of PLA and the mechanical strength and bone-stimulating properties of Mg.

In recent years, there has been growing interest in the development of 3D-printable PLA/Mg composite materials for bone tissue engineering applications. Mg-doped PLA composites have been shown to support the attachment, proliferation, and differentiation of various types of bone cells, such as osteoblasts and osteocytes, in in vitro studies [12]. The Mg content of the composites can affect their biocompatibility, mechanical properties, and proliferation. Mg-doped PLA composites have also been demonstrated to stimulate the formation of new bone tissue in animal studies. These composite materials can be used to create complex and customized bone scaffolds with controlled porosity, mechanical properties, and degradation rates, which can provide a suitable environment for bone tissue regeneration. Additionally, the use of 3D-printing technology enables the fabrication of patient-specific bone implants, which can improve implant–bone integration and reduce the risk of implant rejection. Many conventional methods have been employed for the preparation of magnesium/polymer composite scaffolds, as discussed in detail in our previous review on this topic [13].

However, recent papers on the PLA/Mg composite 3D printing have employed different preparation techniques [14,15]. In this paper, we aim to successfully develop filaments for FDM using a simple solvent evaporation technique. Four PLA + Mg compositions were developed and characterized to assess the effectiveness of the technique and were then used for printing test samples on a low-cost FDM 3D printer.

## 2. Material and Methods

### 2.1. Materials

**Figure 1:**
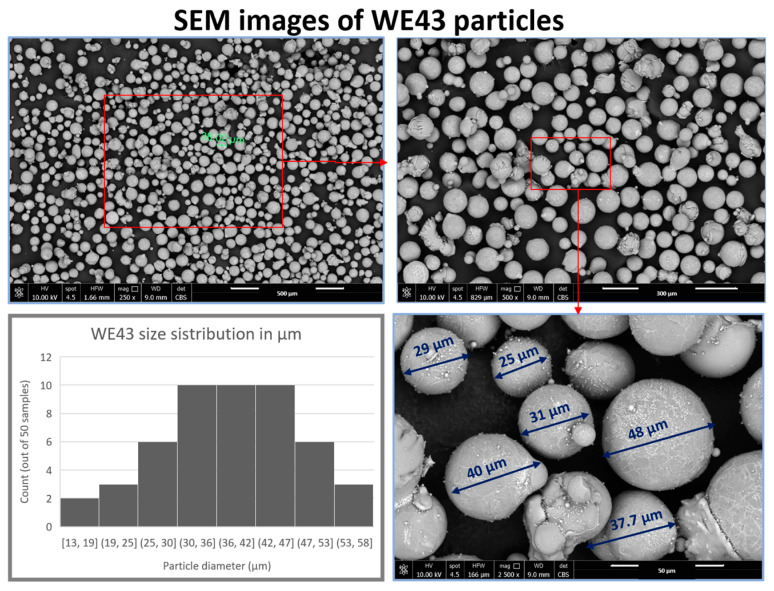
SEM images of WE43 particles and their size distribution.

Polylactic acid (PLA) particles with a nominal granule size of 3–5 mm, a melting point around 170 °C, and an average molecular weight of approximately 193,300 g/mol, were purchased from Goodfellow Cambridge Limited (Cambridge, UK). The density and Melt flow rate (MFR) of the PLA are 1.24 g/cm^3^ and 8, respectively. Mg alloy (WE43, purity 99.95%) powder with an average diameter of 35–50 μm was provided by Nanografi Nanotechnology (Ankara, Turkey) (SEM images shown in Figure 1). Chloroform (Boiling point: 59.5–60.5 °C, density: 1.48 kg/L) was used as a PLA solvent supplied by VWR Chemicals (Hannover, Germany).

### 2.2. Preparing PLA/WE43 Composite Films

The composite films were developed using the solvent evaporation method. PLA pellets and varying amounts of Mg alloy powder (5 wt%, 10 wt%, 15 wt%, 20 wt%) were added to chloroform to obtain a concentration of 125 g/L and mechanically stirred for 24 h at 400 rpm (Table 1). The solution was then poured onto a metal plate, let dry for 24 h at room temperature, and peeled from the metal plate. After the chloroform evaporation, the PLA/Mg composites were taken for characterization.

### 2.3. Characterization of Composite Films

The surface morphology of the composite films was examined using a field emission scanning electron microscope (an FEI Quanta650FEG was used for imaging (FEI, Hillsboro, OR, USA)). The dried samples were cut into suitable sizes as per the holder requirements and their surface morphology was studied. The surface morphology of the composite was examined using a field emission scanning electron microscope functioning at 2 KV accelerating voltage and a working distance of 9.0 mm. The composition of the composite was also analyzed with energy-dispersive X-ray spectroscopy (FEI Quanta650FEG is used for imaging and Bruker Quantax400 for EDS). Thermogravimetric analysis (TA SDT 650, New Castle, DE, USA) was used to determine the thermal stability of the PLA/Mg composites. These composites were cut into pieces with a weight of 10 ± 2 mg and placed in a ceramic crucible. The heating cycle was set to increase from room temperature to 700 °C with a heating speed of 10 °C/min under a nitrogen gas purge. The cold crystallization temperature, melting point, and decomposition temperature of DSC and the remaining mass percentage of TGA at 500 °C were identified from the testing curves. The values for melting temperatures (Tm), enthalpies of melting (Δ*Hm*) and melting temperatures (Td) were analyzed using the TA instruments software package (V4.5A). Δ*Hm*_0_ is the enthalpy of melting for 100% crystallized PLA, which is equal to 93.7 J/g, as seen from the literature, and w is the weight fraction of PLA. The crystallization percentage (*Xc*, %) was estimated using the following equation:

$$Xc\;\%=\frac{\Delta Hm}{\Delta Hm_{0}\times w}\times 100$$

The X-ray diffractometer (XRD) (Bruker D8 Advance, Bruker, Billerica, MA, US) was used to determine the crystal structures of the composites using Cu Kα radiation, within an angle range of 10°–90° at a scan rate of 0.1°/min. Fourier-transform infrared spectrometry (FTIR) was used to detect the functional groups in PLA/Mg composites. FT-IR measurements were carried out in transmittance mode on a Thermo Scientific Nicolet iS50 FT-IR spectrometer (Thermo Nicolet Corporation, Waltham, MA, USA) equipped with an attenuated total reflectance (ATR) sampling accessory with a diamond crystal plate. Spectra were recorded with 32 scans per sample/background in the spectral range of 4000–400 cm^−1^ at 4 cm^−1^ spectral resolution.

### 2.4. Extruding PLA/WE43 Composite Filaments

**Figure 2:**
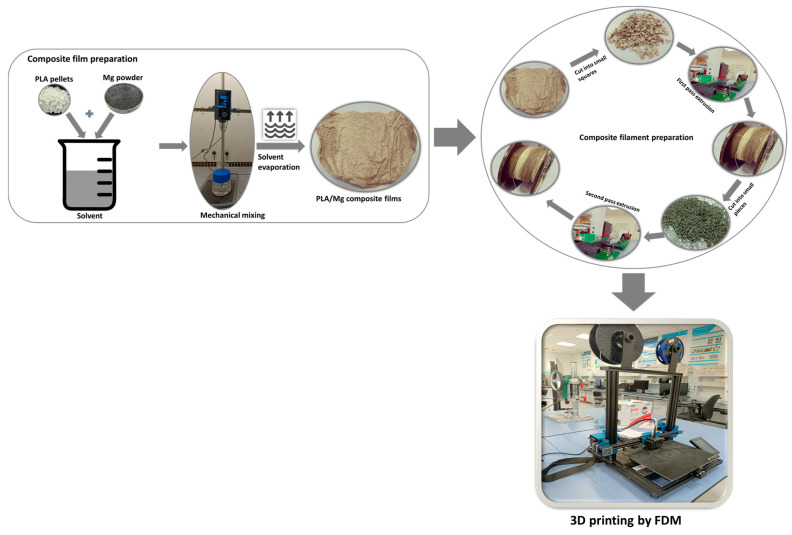
Schematic of the fabrication process.

Figure 2 shows the schematic of the sample preparation procedure employed in this paper. The PLA/WE43 composites of various concentrations prepared above as films were shredded into uniform-sized squares for use as feedstock to the extruder. A Filabot EX2 (Filabot, Vermont, VT, USA) system was used as the most advanced filament extruder. The filaments were extruded through melt blending extrusion at a temperature of 175 °C via a nozzle with a diameter of 0.2 mm. The extruded filament was then shredded into small uniform pieces and fed into the extruder for a second time to obtain homogeneity and consistency in the distribution of Mg particles. These filaments with various concentrations of Mg were then used for further studies and 3D printing.

### 2.5. Characterization of the Composite Filaments

The cross-section morphology of the composite was examined using a field emission scanning electron microscope functioning at 2 KV accelerating voltage and a working distance of 9.0 mm (FEI Quanta650FEG is used for imaging). The filament cross-section was polished with fine-grit-size sandpaper to reveal a uniform surface for the morphology study. This was done to assess how well Mg particles were integrated into the PLA matrix. The surfaces were also subjected to SEM analysis to check for filament flaws such air inclusions.

### 2.6. 3DP of PLA/WE43 Composite Filaments

The manufactured PLA-Mg composite filaments were put into an industrial 3D printer that uses FDM (SOVOL-SV02 by SHENZHEN Lian Dian Chuang Technology LTD, Guangdong, China). The nozzle diameter of the printer was 0.4 mm, and the layer resolution was kept at 0.2 mm. Dog bone designs of ASTM D638-10 Type IV standard and having dimensions 57.5 mm × 9.5 mm × 1.6mm were fed into the 3D printer. The scaffolds were designed using the Solidworks software (2017 SP5, Dassault Systèmes, Paris, France), and were then sliced for 3D printing using Ultimaker Cura slicing software V4.5 (Ultimaker, Utrecht, The Netherlands). The printing temperature was set at 180 °C, the printer bed temperature was set at 60 °C, and the printing speed was kept at 50 mm/s. The heated, loaded composite filaments were extruded through the 3D printer’s nozzle and then laid down layer by layer along a predetermined path according to a computer-aided design (CAD) file. Paper glue was used as an adhesion improver on the build plate for all materials.

### 2.7. Statistical Analysis

All experimental data and 3D printing was carried out three times to confirm the consistency of the reported results. Wherever necessary, the results were reported as mean value ± SD.

## 3. Results and Discussion

### 3.1. Surface Morphology Study

**Figure 3:**
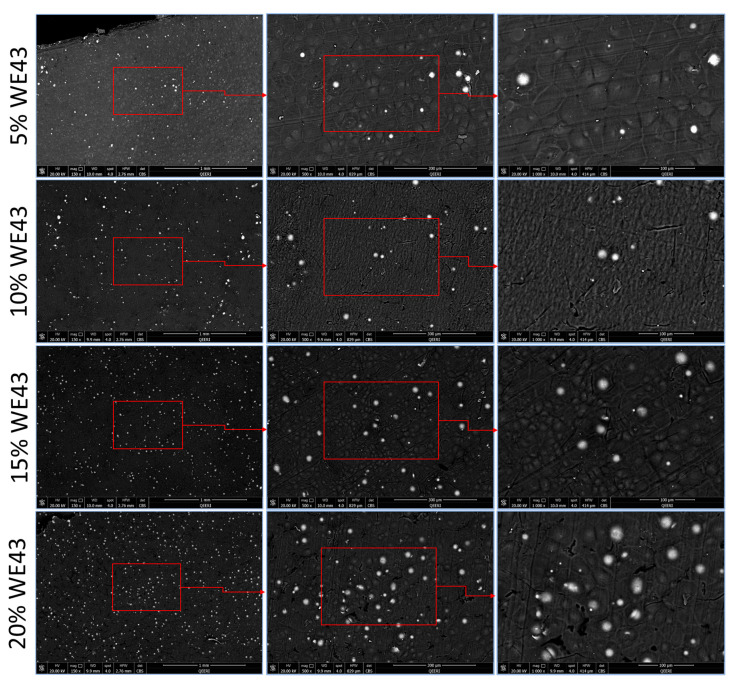
SEM images of the composite films. The figure shows the SEM images of the various compositions of Mg/PLA composite films developed at different magnification levels.

To understand the morphology of the developed films, an SEM examination was carried out. The surface morphology of the film will help us understand how the final 3D-printed surface would look and behave, which will in turn influence the cell attachment and proliferation. The images were also studied to understand the distribution of magnesium particles in the matrix. Since all the films were continuous and defect-free, it was clear that the preparation technique was effective. The SEM images of various WE43 percentages in the PLA matrix are depicted in Figure 3. All the images show the presence of WE43 magnesium particles distributed uniformly throughout the area under study. This indicates that the preparation technique has led to the homogenous mixing of the magnesium particles in the PLA matrix in the various blend ratios. As can be seen in these images, the Mg particles are largely uniform and evenly dispersed throughout the PLA matrix, suggesting the mixing method’s potential effectiveness in producing high-quality polymer/metal composite filaments. In turn, this would facilitate the extrusion of filaments with uniform distribution for flawless 3D printing. The examined films have a rough surface, which is made even rougher by the integration of a higher magnesium content as a result of the presence of magnesium on the surface. The evaporation of chloroform has led to the formation of grain-shaped features on the surface of the films. This can be attributed to the availability of more nucleation sites in the presence of a higher number of magnesium particles by the particle simulated nucleation (PSN) phenomenon. In particulate-reinforced materials, recrystallization by particle-stimulated nucleation generally dominates during thermomechanical processing. This can be seen in the literature, where different reinforcements in the polymer matrix have led to the observance of this phenomenon [16,17,18]. The increase in the presence of nucleation sites limits the growth of the grains in the composite leading to cracks/voids on the surface as is evident in the images of higher Mg compositions.

**Figure 4:**
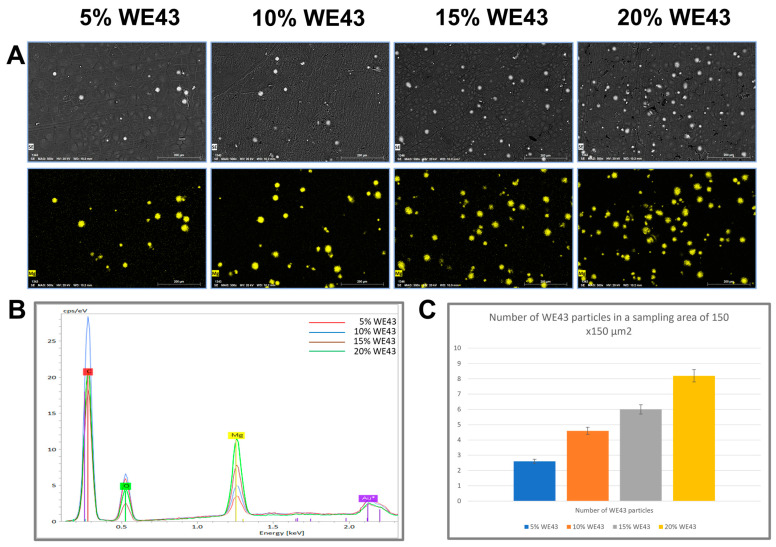
EDS mapping of magnesium dispersion on the various composite films: (**A**). EDS mapping showing the magnesium particles (**B**). EDS plot showing the peaks of magnesium in the various concentrations (**C**). Plot showing the average number of magnesium particles in a sampling area in the EDS images.

Figure 4 shows the EDS mapping images of the various compositions of PLA/WE43. As the concentrations of WE43 increases, the number of magnesium particles visible across the surface also increases. The magnesium particles are uniformly distributed in all cases, and the number of particles increases with increasing concentration of WE43. The increase in the content of WE43 in the blends can be seen to be validated by the similar linear relationship in the average particles in a random sampling area of the EDS images.

### 3.2. XRD and FTIR Results

**Figure 5:**
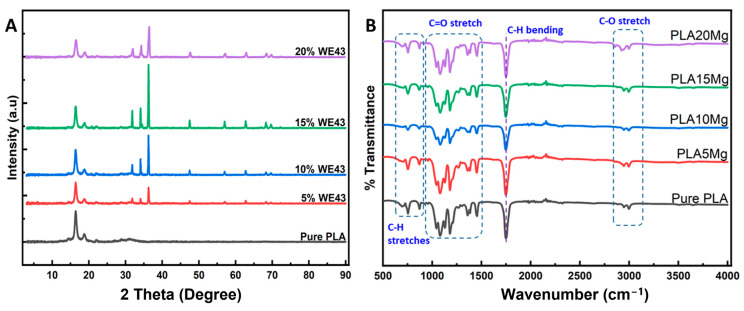
(**A**) XRD characterization of the pure and composite films. (**B**) FTIR characterization of the pure PLA and composite films.

The XRD spectra of PLA and Mg-incorporating samples are shown in Figure 5A. As the concentration of Mg increased in the PLA samples, the peaks in the Mg alloy at 2θ = 32°, 34°, and 36° became more pronounced. The pure PLA had a semi-crystalline structure, as indicated by peaks at 2θ = 16° and 19.5°, which correspond to the “α” crystallization phase. With increasing Mg concentration, the XRD pattern showed less intense peaks, indicating a lower degree of crystallinity for the Mg-loaded samples. It is important to note that the XRD peaks only provide information about the crystallinity of a thin surface layer and the bulk crystallinity may be different. These results are in agreement with similar studies reported in the literature [19].

FTIR spectroscopy is a method used to identify the chemical functional groups in a material by measuring the absorption of infrared radiation. Figure 5B shows the FTIR spectra of the PLA/Mg composite, which displays the absorption bands of the functional groups present in the PLA matrix and the Mg reinforcing particles. The minor differences between PLA and PLA-Mg composites suggest that the chemical linkages were similar and consistent with the literature [20]. The C-H stretch shows a slight shift in position, but overall, there is no major change in the peak positions when Mg particles are incorporated. However, there is a slight decrease in peak intensity. In the FTIR plot, the following characteristic peaks may be seen:

- Bands caused by the CH group’s symmetrical and asymmetrical stretching vibration, measuring between 2900 and 3000 cm^−1^;
- Bands allocated to the C-O-asymmetrical Cs and symmetrical stretching vibrations between 1000 and 1200 cm^−1^;
- Bands assigned to the CH_3_ group’s stretching vibration and bending vibrations between 1300 and 1500 cm^−1^;
- Bands caused by the C=O group’s stretching vibration start to show at about 1748 cm^−1^.

Additionally, no new absorption peaks or changes in peak positions were observed. This indicates that the magnesium particles blend well with the polymer matrix. However, there is no indication of a chemical reaction between the PLA and the magnesium particles during the blending process.

### 3.3. Crystallization and Thermal Degradation Behavior

**Figure 6:**
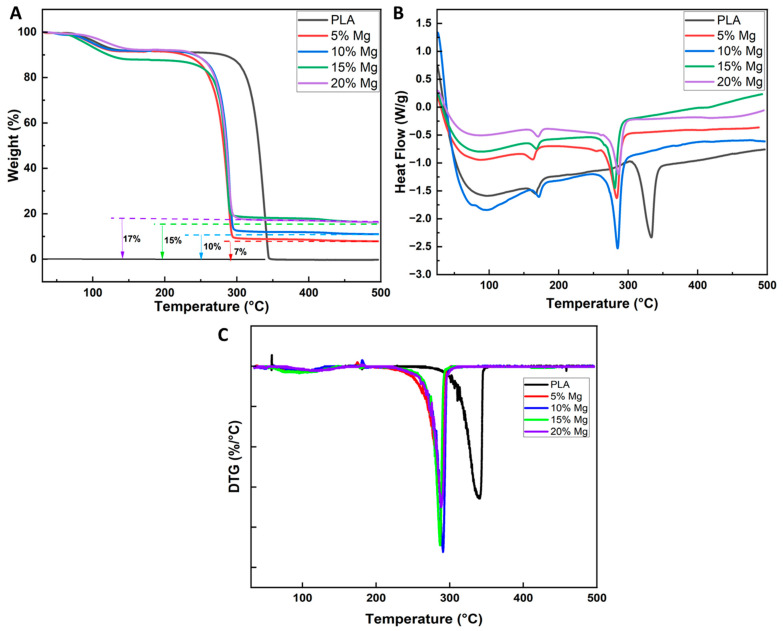
(**A**) TGA characterization of the PLA and composite films, (**B**) DSC characterization of the PLA and composite films, (**C**) DTG curves of the PLA and composite films.

TGA is commonly used to determine the thermal stability of a material, as well as its decomposition temperature and the weight loss resulting from thermal decomposition. In the case of polylactic acid (PLA), TGA can be used to determine the temperature at which the material starts to degrade and the extent of degradation that occurs at different temperatures. According to Figure 6A,C, an initial mass loss was observed for all samples after 70 °C, most likely because of the solvent evaporation. PLA degrades thermally between 300 °C and 370 °C, whereas PLA/Mg composites start to degrade sooner and do so between 250 °C and 300 °C. Mg addition speeds up heat breakdown in the PLA. In contrast to pure PLA, which was entirely decomposed, the composite mixes also showed a residue quantity. This is to be expected, as the remaining residue can be associated with the magnesium content. The residual amount values associated with magnesium are close to the original magnesium wt% in the composite samples as shown in Figure 6A. The optical microscopy study of cross-sections revealed that Mg particles are evenly distributed within the PLA/Mg composites (Figure 3 and Figure 4). It is clear from these results that Mg incorporation speeds up the degradation of PLA when subjected to elevated temperature. When the polymer is exposed to high temperatures, Mg acts as a catalyst for the PLA depolymerization reaction. Similar thermal analysis results have been reported in the literature [21,22].

To examine the thermal behavior of PLA/Mg composites in the processed blends, DSC measurements were taken. The thermal properties of PLA and PLA-Mg composites are presented in Figure 6B. The data observations obtained from the plots has been tabulated in Table 2. Three thermal transitions are observed in all samples, independently of the presence of Mg particles. The first transition with increasing temperature is an endothermic step associated with the glass transition of PLA (Tg). It should be noted that the Tg for pure PLA is around 43 °C. On the other hand, the addition of Mg has clearly increased the Tg to a maximum of 52 °C for 10% Mg samples. All loaded samples showed Tg around this value. The increase in Tg with increasing filler content might be due to the polymer wetting the surface of the Magnesium particles, as reported in the literature [23]. Additionally, PLA presented a melting peak (Tm) at 167.8 °C. The Tm peak increases with increasing Mg content, reaching a maximum of 172.8 °C for 20% Mg samples.

The characteristics of PLA, such as the melting temperature and decomposition temperature, can be seen and compared in all the samples (Table 2). These data suggest that Mg particles have little effect on the PLA melting peaks. However, the presence of Mg particles serves as a nucleation agent, causing heterogeneous nucleation in the samples. Zerankeshi et al. reported similar findings when using graphite particles in the PLA matrix [24,25]. The melting enthalpies also show little variation among the Mg-loaded samples, but showed significantly lower values when compared to no filler PLA samples. PLA samples showed crystallinity of around 35%, which shows that the polymer used was in a semicrystalline state. The study of PLA crystallization from melt has been reported extensively in the literature. It is well known that the rate of crystallization of PLA from melt is slow, thus leading to crystallization of a tiny fraction of PLA [26,27]. Incorporating Mg particles led to the drop of crystallinity to lowest value of 12% for 20% Mg samples. The intensity of the crystallization peaks is unaffected by the varying the Mg concentration; therefore, there were no dramatic variations in the degree of crystallinity. Therefore, it may be argued that varying the concentration of Mg particles in the PLA matrix has no impact on the crystalline structure of loaded samples. When creating materials for biomedical uses, this is crucial to keep in mind, because alterations in chemical composition might have negative effects on the body, like potent immunological reactions.

### 3.4. Characterization of the Extruded Filaments

**Figure 7:**
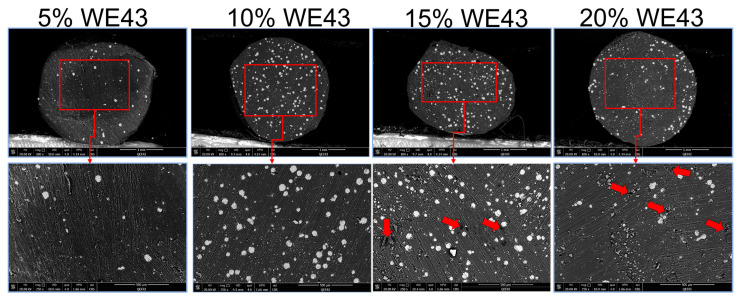
SEM images of the cross-section of extruded filaments of various concentrations of composites.

It was feasible to create PLA filaments with progressively increasing Mg contents of 5, 10, 15, and 20% (wt). For each PLA-WE43 composition, melts were extruded from the extruder and monitored in real time over a period to determine when the desired filament diameter of d = 1.75 ± 0.25 mm was reached. Only filaments in this diameter range can be used in the SOVOL SV02 printer. We evaluated the process stability over time by tracking the divergence in filament diameter from the filament extruder by continuous monitoring. The non-uniform extruded filaments were again chopped into little pieces and extruded a second time to enhance the quality. This made it easier to prepare filaments for feeding into the printer with regular lengths and uniform diameters. Micrographs using scanning electron microscopy showed that the Mg particles within PLA-Mg filaments were distributed uniformly (Figure 7). The examined filaments have a rough surface, which is made rougher by incorporating magnesium. The Mg particles are comparatively uniform and were evenly dispersed throughout the PLA matrix, as seen in this picture, indicating the mixing method’s potential effectiveness for producing high-quality polymer/metal composite filaments. However, in composite filaments with an Mg concentration of more than 10 wt%, pores can be detected close to the filler particles. This might result from the magnesium particles’ reduced flowability and the accumulation of numerous clustered magnesium particles on the composite’s outer surface. The best distribution is shown by the 15% WE43 samples, although the printability may suffer due to printing disruptions because of the pores seen in the cross-section. Due to the uneven distribution of magnesium particles and the increased prevalence of pores, 20% WE43 samples display undesirable characteristics. This result might be explained by the higher volume fraction of Mg particles in the molten polymer, which is related to increased viscosity. Higher solid contents result in a typical increase in viscosity that makes it challenging to extrude filaments as mentioned in the literature [28]. During the extrusion process, the Mg particles could prevent the PLA matrix from flowing freely, creating micro voids close to the Mg fillers (marked with red arrows). According to the reported data, increasing the Mg content in the PLA-Mg composite filaments causes Mg particles to cluster and develop pores around them, which can all have an impact on the qualities of printed items. These results support those reporting in the previous literature, where similar findings were observed using a similar experimental design [15].

### 3.5. Printability of the Filaments

The molten material must support the weight of the printed layers in addition to flowing through the nozzle without clogging formation at the nozzle tip for successful 3D printing of PLA/Mg composites using the FDM process. In order to determine whether filaments are appropriate for creating intricate geometrical features and whether they can maintain structural integrity during the printing process, samples of the various PLA-Mg compositions were 3D printed. As discussed in the previous section, the DSC curves show that the melting temperature of all the compositions was around 175 °C. Hence, this temperature was selected for the printing of all the compositions. The nozzle temperature was set at 175 °C, a printing speed of 50mm/s was used, and the bed temperature was kept at 60 °C for the printing process. All the printing parameters are summarized in Table 3.

**Figure 8:**
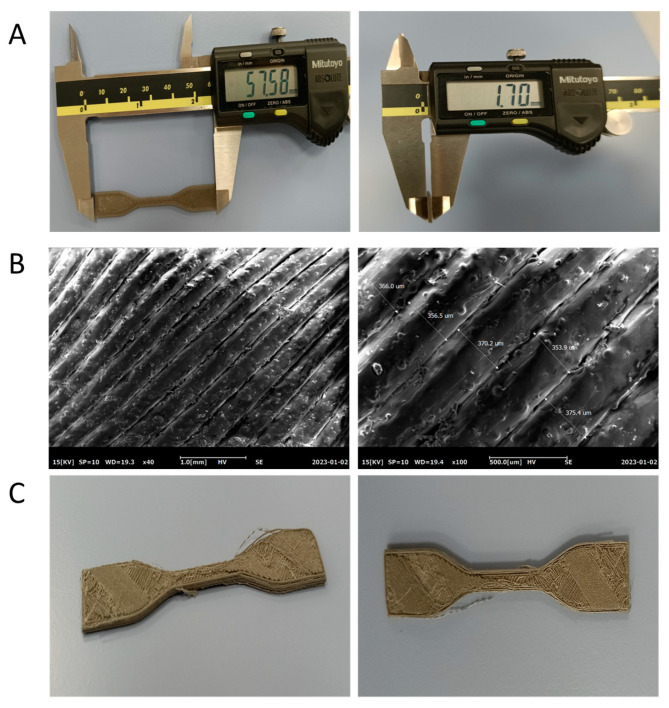
3D printing of the composite filaments: (**A**) Images show the prepared dog bone-shaped specimens of 10% WE43 composition. (**B**) SEM images showing the surface morphology of the printed specimens shown in (**A**). (**C**) Defective 3D-printed parts for the 15 and 20% WE43 composition.

It was found that 5% WE43 and 10% WE43 composite filaments were easily printable with no visible defects or inconsistencies. As shown in Figure 8A, the printed parts met the input design parameters without any significant deviance from the input CAD file. In addition, the finish was also quite smooth, without any gaps in the layers. This can be attributed to the filaments showing no pore formation in the SEM cross-section images as discussed in the previous section. The SEM images in Figure 8B show that the layers are smooth and consistent, and do not show any disruptions on the printed surface. However, the 15% WE43 and 20% WE43 samples were not printed effectively, owing to the increase in the presence of the pores in their filaments. The dog bone samples printed from 15% and 20% WE43 showed visible deviations from the input design, as well as many inconsistencies, as shown in Figure 6C.

## 4. Conclusions

This study investigated the development of Mg-filled PLA filaments as a new composite material for 3D printing. It presented the viability of these PLA/Mg composites for extrusion into filament form and fabrication into test samples using FDM-based 3D printing. The influence of Mg inclusion on the thermal, physicochemical, and printability behavior of PLA was evaluated. The SEM study of the composite films showed that the magnesium particles were uniformly distributed in all of the compositions, and the number of particles increased with increasing concentrations of WE43 particles. No new absorption peaks or changes in peak positions were observed in the FTIR results, indicating that the magnesium particles blended well with the polymer matrix, and that there was no chemical reaction between the PLA and the magnesium particles during the blending process. The thermal studies showed that the addition of Mg increased the glass transition temperature, reaching a maximum of 52 °C for the 10% Mg samples. Additionally, the melting peak increased with increasing Mg content, reaching a maximum of 172.8 °C for the 20% Mg samples. Incorporating Mg particles led to a drop in crystallinity to a minimum value of 12% for the 20% Mg samples compared to the 38% of the PLA samples. However, there were no dramatic variations in the degree of crystallinity among the Mg-loaded samples. The filament cross-section images show that the distribution of magnesium particles is uniform until 15% WE43 concentration. Subsequently, the distribution was non-uniform, and the magnesium particles were pushed toward the periphery of the filament cross-section. Additionally, the presence of pores in the vicinity of the magnesium particles when increasing from 15% WE43 to 20% WE43 affected their printability. It was observed that 5% and 10% WE43 composite filaments showed good printability when using the low-cost FDM 3D printer. Therefore, they have potential to be used as composite biomaterials for 3D printing of bone implants. Further research needs to be carried out to assess the mechanical and biological properties of these composites.

## Figures and Tables

**Table 1:** Compositions of prepared PLA/Mg composites.

Material	Composition (wt%)	
	PLA	Mg (WE43)
PLA	100	00
5% WE43	95	05
10% WE43	90	10
15% WE43	85	15
20% WE43	80	20

**Table 2:** Glass transition temperature (Tg), degradation temperature (Td), melting temperature (Tm), melting enthalpy (ΔHm), and crystallinity (X) data of the samples obtained from the DSC thermograms.

Sample	Tg (°C)	Td (°C)	Tm (°C)	ΔHm (J/g)	X (%)
PLA	43.2	333.41	167.81	35.29	37.91
5% Mg	49.3	295.69	171.82	13.11	14.08
10% Mg	51.6	289.12	172.76	14.15	15.20
15% Mg	48.25	289.14	169.5	16.43	17.65
20% Mg	50.46	281.3	171	12.06	12.95

**Table 3:** Table showing all the printing parameters used for the FDM process.

Printing Parameters	
Nozzle temperature	175 °C
Printing speed	50 mm/s
Printing bed temperature	60 °C
Layer thickness	0.2 mm
Nozzle diameter	0.4 mm

## Data Availability

The data presented in this study are available on request from the corresponding author.
